# Assessment of Behavioral, Clinical, and Histological Outcomes in Sprague-Dawley Rats Housed in Enriched Colony Cages Versus Conventional Pair Housing over 28 Days

**DOI:** 10.3390/ani15243525

**Published:** 2025-12-07

**Authors:** Arman Shamsi, Peter Niebl, Annika Kalina, Stephanie Krämer, Marc W. Nolte, Simone Tangermann

**Affiliations:** 1 CSL Innovation GmbH, Emil-von-Behring-Str. 76, 35041 Marburg, Germany; arman.shamsi@cslbehring.com (A.S.); peter.niebl@cslbehring.com (P.N.); annika.kalina@cslbehring.com (A.K.); marc.nolte@cslbehring.com (M.W.N.); 2 Laboratory Animal Science and Animal Welfare and Interdisciplinary Center for Animal Welfare Research and 3Rs (ICAR3R), Justus Liebig University Giessen, Frankfurter Strasse 110, 35392 Giessen, Germany; stephanie.kraemer@vetmed.uni-giessen.de

**Keywords:** refinement, rat, colony housing, enrichment, drug development, safety assessment

## Abstract

The standardization of husbandry in animal studies for drug development aims to minimize variability and enhance inter-laboratory comparability. Rats are a commonly used species in such studies. This standardization yields housing conditions that do not reflect the natural environment of rats, raising animal welfare concerns and prompting discussions about whether such conditions might also influence physiological or behavioral outcomes, thereby potentially affecting the translatability of study results. In this study, we compared a two-level, enriched colony cage housing groups of ten Sprague-Dawley rats with conventional pair-housed Type IV cages over 28 days, asking whether social and physical complexity alters parameters relevant to early drug development and safety assessment. These included body weight, food and water intake, behavior (open field, elevated plus maze), hematology, clinical chemistry, blood gases, gross pathology, organ weights, histopathology, and IgG levels. Differences in key measures between housing conditions were minimal: Hematology showed time-restricted shifts. On study day 28, immunoglobulin G concentrations were higher in conventionally housed animals, though values were comparable and within the normal range. Behavioral tests revealed notable but limited differences between housing conditions, mainly involving locomotor activity. Clinical chemistry and blood gases were largely unchanged. Body weight, food and water intake, and pathology parameters were comparable. Together, these findings demonstrate that enriched colony housing can be implemented as a refinement strategy without compromising the integrity of key study endpoints relevant to early drug development and safety assessment.

## Simple Summary

Laboratory rats are usually housed in small standard cages to reduce variability, but these conditions do not reflect their natural environment and prevent natural behavior patterns. We tested whether housing Sprague-Dawley rats into larger, two-level “colony” cages (10 rats/sex/cage) changed results that matter for early drug development. Over 28 days we measured body weight, food/water intake, behavior (open field, elevated plus maze), hematology, clinical chemistry, blood gases, organ weights, histopathology, and immunoglobulin level. Overall, housing had little impact. Blood counts showed brief, time-limited shifts. By day 28, conventionally housed rats had slightly higher immunoglobulin G, but values remained comparable and within normal limits. Behavioral tests showed a few differences, mainly greater locomotor activity in standard cages. Clinical chemistry and blood gases were largely unchanged. Body weight, food and water intake, and gross and microscopic pathology were comparable between groups. In conclusion, our study shows that increasing animal welfare by housing rats in larger cages with more enrichment is feasible without compromising data relevant to drug development.

## 1. Introduction

Animal experiments have played a pivotal role in the research and development of drugs, chemicals, and medical devices [1]. Historically, there has been a strong trend toward standardizing conditions in animal experiments, both in biomedical research and drug development, with the aim of enhancing reproducibility, minimizing biological variability, and ensuring regulatory compliance. In addition to genetic standardization, efforts have focused on harmonizing hygiene management and housing conditions to reduce variations in data and improve data consistency, reduce variability, and facilitate comparability across studies and laboratories [2]. In early discovery studies, formal guidelines for housing laboratory animals are often absent. Nonetheless, pharmaceutical and chemical companies, as well as contract research organizations, frequently rely on internal documents—such as standard operating procedures—to define housing conditions. These internal standards often mirror those found in regulatory guidelines, ensuring reliability and animal welfare even in exploratory phases.

Existing toxicology guidelines and study protocols (e.g., OECD, EPA, FDA, ICH/EMA, EFSA) historically recommend housing rats in small groups of the same sex [2,3,4,5]. For example, OECD Test Guideline 407 specifies that no more than five rats should be housed per cage. Such guidelines reflect concerns that overcrowding might provoke aggression, elevate stress, complicate individual monitoring, and increase data variability [3]. Indeed, substandard or overly dense housing is known to lead to inter-animal aggression, stress-induced stereotypes, and heightened anxiety [6]. To minimize these risks under conventional cage conditions, regulators have traditionally limited group sizes to just a few animals per standard cage. Exposure of animals dosed with chemicals via food or drinking water can also be more easily monitored in small groups. This type of housing contrasts sharply with the natural living conditions of rats. In the wild, rats are highly social animals that live in large colonies with complex group structures, overlapping generations, and dominance hierarchies. This social organization is closely tied to their spatial ecology: they construct extensive burrow systems and rely on complex, structured space for resting, refuge, exploration, and social interaction [7]. Consistent with their natural history, laboratory rats are strongly motivated to access structural resources, shelters and nesting materials [8], vertical space to stand upright, and substrates for burrowing, and when given choices they prefer more complex, structured cages over barren ones [9]. Accordingly, enriched housing, combining environmental and social opportunities, enhances cognitive performance, motor function, social behavior, and affective state, while markedly reducing stereotypies and other abnormal behaviors, consistent with an overall protective effect on welfare [10]. The strength of rats’ social motivation is especially clear in operant choice studies: even after extended drug access that produces addiction-like taking, rats overwhelmingly choose interaction with a conspecific over self-administering heroin or methamphetamine [11]. Notably, prevailing guidelines rarely consider the use of larger, environmentally and socially enriched enclosures that could safely accommodate much larger social groups. Standard laboratory rat cages with approximately 1820 cm^2^ of floor space (as defined by the minimums in Annex III of EU Directive 2010/63) are sufficiently small that animals cannot display many natural behaviors: rats cannot run, burrow, or climb in these confined spaces [12]. Such spatial restriction intrinsically compromises welfare and can induce stress and abnormal behaviors [13]. By contrast, when rats are provided with cages that substantially exceed standard dimensions—ideally with multiple levels and vertical complexity—they can be housed in larger groups without crowding or conflict.

Nowadays, scientific and ethical concerns increasingly challenge current practices in drug development. The 3Rs principle—replacement, reduction, and refinement—first introduced by Russell and Burch in 1959, is gaining renewed importance and prompting a re-evaluation of established procedures [14]. Examples of changing regulatory practices include a reduction in the number of control animals through virtual control groups [15], the implementation of in vitro and in silico approaches, as well as literature-based weight-of-evidence strategies [16]. Scientific concerns regarding standardized housing include limited translatability to humans and reduced data robustness. Data collected under minimally enriched conditions may reflect psychological stress, potentially compromising relevance for translational research [13].

The aim of this study is to investigate whether enriched housing conditions, implemented through the use of specially designed colony cages, may influence outcomes relevant to early drug development and safety studies for drug candidates and chemicals. We examine endpoints commonly assessed during safety studies, including hematology, clinical chemistry, pathology, and animal behavior. The study design follows a 28-day repeated-dose toxicology protocol, as shorter study durations are also often appropriate for early pharmacodynamic and pharmacokinetic investigations in disease models. Additionally, the 28-day study typically represents the first formal step in a regulatory safety program following dose-range-finding studies. In addition to evaluating biological outcomes, we describe the development and implementation of colony cages, and assess the time and effort required for animal care under both conventional and enriched housing conditions. Finally, we discuss the potential integration of enriched housing concepts—such as colony cages—into the broader drug development process, considering scientific, ethical, and laboratory operational implications.

## 2. Material and Methods

### 2.1. Animals

Forty Hsd:Sprague-Dawley rats aged 41 ± 5 days were purchased from Envigo (Envigo RMS GmbH C/O, Venray, The Netherlands). After the arrival of the animals in the facility, the male rats underwent 9 days of acclimatization and female rats 8 days. To individually identify animals, subcutaneously implanted TP500 microchips (TP500, Temperature transponder, Bio Medic Data Systems, Waterford, WI, USA) and non-toxic permanent markers (green and blue) were applied on the tails of the animals for visual identification.

All animals were housed under controlled conditions (temperature 22 ± 2 °C, relative humidity = 50 ± 10%, light/dark cycle = 12 h/12 h, light intensity = 200 lux during light cycle and 400 lux during the activity time of laboratory staff) with free access to water and food (maintenance diet, ssniff Spezialdiäten GmbH, Soest, Germany, product number: V1534-703). Environmental enrichment included polycarbonate tunnels (two per floor; 155 mm length × 75 mm diameter; ZOONLAB GmbH, Animal Husbandry Experts, Castrop-Rauxel, Germany) and Fat Rat Hut shelters (polycarbonate; one per floor; 150 × 165 × 85 mm, L × W × H; ZOONLAB GmbH, Animal Husbandry Experts, Castrop-Rauxel, Germany).

Two water bottles were installed on each floor, resulting in a total of four bottles per cage. Animals (*n* = 40) were randomly allocated to the two housing conditions (conventional and colony housing) using pre-assigned subject identifiers. Randomization was stratified by sex and body weight to ensure these factors were balanced across treatment groups. The randomization key was generated prior to the study beginning.

### 2.2. Housing Conditions

**Figure 1:**
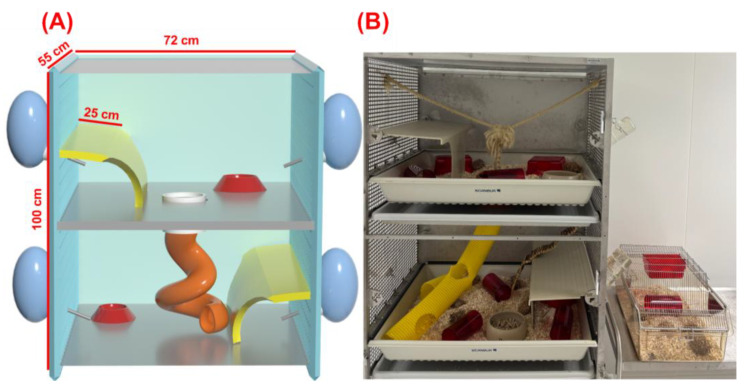
Colony housing versus conventional housing used in this study. (**A**) Schematic of the custom two-level colony cage built on a Scanbur EC3 frame. Each level has an internal floor of 71.5 × 54.5 cm (3896 cm^2^); the levels are connected by a central opening (Ø 16 cm) fitted with a vertical tube. The opening reduces the floor area by 201 cm^2^, yielding 7592 cm^2^ of usable floor area across the two connected levels. Internal height per level is ~50–51.5 cm; plateaus create sheltered zones above the floor. Each level contains two polycarbonate tunnels and one shelter (“Fat Rat Hut”); food was available on both levels and water was provided by two bottles per level (four per cage). (**B**) Representative views of the colony cage: Interior (showing the vertical tube, climbing rope, and flexible bridge enabling movement between levels and providing vertical complexity) and room-level side-by-side with a conventional cage for scale. In the study, rats were housed as groups of 10 (same sex) in colony cages and as pairs in conventional cages; environmental conditions and vendors are detailed in Materials and Methods.

The animals were housed in two different housing conditions in the study: Colony cages or conventional cages (Figure 1). The colony cages were designed based on the Scanbur EC3 cage (Scanbur, Karlslunde, Denmark). The middle and upper levels were connected through a central opening with a radius of 16 cm. A pipe with a diameter of 16 cm was inserted. A rope was installed to enable rats to climb between the two floors. Two plateaus of the original rabbit cage were installed to allow additional levels. The total floor area of each level was 3896 cm^2^. The opening in the floor resulted in a loss of floor area of 201 cm^2^. Thus, the two connected levels had a total floor area of 7592 cm^2^. Ten animals were housed in the colony cage for the study.

The conventional cages used were from Tecniplast (EUROSTANDARD Type IV S, model 1354G; 59.8 × 38 × 20 cm; base area: 1820 cm^2^; equipped with raised wire lids Series 117/120, Tecniplast, Hohenpeißenberg, Germany). The animals were housed in pairs in the conventional cages and as a group of 10 rats (same sex) in the colony cages. All experiments were approved by the responsible animal ethics authority (Regierungspraesidium Giessen) and performed in accordance with the animal ethics license number G 79/2022.

Immediately after transfer to the colony cages, all rats were continuously observed for 30 min by the attending veterinarian together with a trained animal-care technician as part of the facility’s standard clinical observation protocol after transferring animals to new housing conditions. The primary purpose was to detect potential injuries, pain or distress, and early signs of social instability and other welfare-relevant abnormalities that would require intervention. On the same day, animals were checked periodically during the remainder of the light phase to observe their general condition, social interactions, and unobstructed access to food and water bottles on both cage levels. To minimize disturbance to circadian activity, an additional 30 min observation was conducted during the first dark phase (active period) by the veterinarian under red light. Across all observations, particular attention was paid to any signs of aggression or exclusion that could limit access to food, water, or either level of the cage.

### 2.3. Experiment Design

The study design is based on a 28-day repeated-dose toxicology study, with some modification for better monitoring of the effects of the two different housing conditions. On days 0, 7, 14, 21, and 28 of the study, 150 µL of blood was sampled from the lateral tail vein. Due to a construction defect in one cage, two male rats sustained minor, superficial tail injuries at the study onset, which was scored according to the scoresheet defined by the animal ethics license. No blood sampling was performed on study days 7, 14, or 21 of these animals. On study day 25, the Elevated Plus Maze test (EPM) was performed, and on study day 26 the Open Field Test (OFT) was performed. Ten animals were sacrificed on day 28 and ten animals on day 29 of the experiment. All animals were monitored daily, and their body weights were recorded. At weekends, the animals were only monitored, without recording the body weight. The group size of 10 animals per group was chosen because it reflects common practice in studies conducted for drug development and safety assessment. In 28-day repeated-dose toxicity studies, regulatory guidelines require groups of 5 animals [17]; in such cases, two groups can be housed in one cage. In contrast, for 90-day repeated-dose studies, guidelines specify group sizes of 10 animals [5]. A group size of 10 animals per group is also relevant for pharmacokinetic and pharmacodynamic study settings. The number of animals per group should always be reduced to the minimum required. We assumed that slight deviations from a group size of 10 animals would not affect the investigated study endpoints.

### 2.4. Behavioral Tests

The OFT and EMP tests were performed under a controlled environment, with sound and light isolation, with a light intensity of 15 lux.

#### 2.4.1. Open Field Test (OFT)

The OFT indexes anxiety-like behavior via rodents’ natural thigmotaxis (preference for the periphery/avoidance of the center) while simultaneously providing measures of general exploration and locomotion in a novel arena. Typical readouts include time in central vs. peripheral zones (less center time indicates higher anxiety), total distance traveled and/or velocity (to detect sedation, hyperactivity, or other central nervous system [CNS] effects), and rearing, which serves as an index of exploratory drive.

The Open Field for Rats (Noldus Information Technology, Wageningen, The Netherlands), with dimensions of 1 m × 1 m, was utilized. The central area of the open field was defined as the central 50 cm × 50 cm, which was equivalent to 25% of the total box area. Animals were placed in open field box facing the lower right corner and were allowed to explore the area for 10 min. Behavior was recorded using an iPhone 15 (Apple Inc., Cupertino, CA, USA), which was mounted externally and operated remotely to ensure that no experimenter was present in the testing room during the recording. The following parameters were evaluated via Ethovision XT version 18.0.1832 (Noldus Information Technology, Wageningen, The Netherlands): Total distance moved (in cm), amount of time spent in the central and peripheral areas of the box, number of entries into central area, and rearing on the wall. For all zone-based measures in the Open Field, zone occupancy and transitions were defined using an “all-body-points” criterion: A rat was considered to be in a given zone only when the three tracked body points (nose, central body point, and tail base) were simultaneously located within the boundaries of that zone on the same video frame. Entries into (and exits from) a zone were time-stamped at the first frame in which all three points crossed the corresponding boundary.

#### 2.4.2. Elevated Plus Maze Test (EPM)

The EPM assesses anxiety-like behavior based on rodents’ innate preference for closed, protected arms and avoidance of elevated open arms. Increased time spent in, and entries into, the open arms reflect reduced anxiety. As in the OFT, total distance traveled and/or velocity help identify sedation, hyperactivity, or other CNS effects, and rearing provides an additional index of exploratory drive.

The EPM for Rats (Noldus Information Technology, Wageningen, The Netherlands) used consisted of two open arms and two closed arms (length of each arm: 52 cm, elevated 60 cm from floor). The animal was placed in the center of the EPM and facing one of the open arms and away from the experimenter. They were allowed to explore for 10 min. Behavior was recorded using an iPhone 15 (Apple Inc., Cupertino, CA, USA), which was mounted externally and operated remotely to ensure that no experimenter was present in the testing room during the recording. The following parameters were evaluated via Ethovision XT version 18.0.1832 (Noldus Information Technology, Wageningen, The Netherlands): Time spent in each arm (open and closed), number of entries into each arm (open and closed), and total distance moved in each arm. In the EPM, the open arms, closed arms, and central platform were defined as non-overlapping zones, and the same all-body-points criterion (nose, central body point, and tail base simultaneously within the zone polygon) was used to determine arm occupancy, entries, and exits. In addition, predefined “investigation zones” within the open arms were scored using a nose-only criterion: An entry was registered when the nose point alone crossed the investigation-zone boundary (irrespective of central body or tail base position) and the number of entries was analyzed accordingly.

### 2.5. Clinical Chemistry and Blood Gases

Clinical chemistry parameters were measured from serum obtained from terminal blood samples collected from the vena cava of each animal. Blood was collected into Minicollect tubes (0.5 mL/0.8 mL CAT Serum Sep Clot Activator, 50 pcs; Greiner Bio-One GmbH, Kremsmünster, Austria) and processed according to the manufacturer’s instructions. Serum samples were analyzed using a Roche Cobas C111 System (Roche Diagnostics GmbH, Mannheim, Germany). The following parameters were determined using assay kits from Roche Diagnostics GmbH, Mannheim, Germany: Total cholesterol (Cholesterol Gen.2 Kit), urea (Urea/BUN Kit), creatinine (Creatinine plus ver.2 Kit), total protein (Total Protein Gen.2 Kit), albumin (Albumin Gen.2 Kit), alanine aminotransferase (ALT, ALTL Kit), aspartate aminotransferase (AST, ASTL Kit), alkaline phosphatase (ALP, ALP2S Kit), calcium (Calcium Gen.2 Kit), phosphate (Phosphate ver.2 Kit), and triglycerides (TRIGL Kit).

Following terminal sampling on day 28, the following parameters (blood gases, electrolytes, and metabolites) were measured from fresh whole blood collected from the vena cava and analyzed immediately using the epoc^®^ Blood Analysis System (Siemens Healthineers AG, Eschborn, Germany) with BGEM single-use test cards (Siemens Healthineers AG, Eschborn, Germany). After the card’s automated calibration, ≥92 µL of whole blood was applied, and the sample type was recorded as venous whole blood. The following parameters were reported: pH, pCO_2_ (mmHg), cHCO_3_^−^, Na^+^, K^+^, Ca^2+^, Cl^−^, cTCO_2_ (mmol/L), glucose (mg/dL), and lactate (mmol/L).

### 2.6. Hematology

Hematology parameters were measured directly from fresh EDTA-anticoagulated blood collected from the lateral tail vein on days 0, 7, 14, 21, and 28 of the study. Blood samples were drawn into tubes containing 10% EDTA anticoagulant, prepared by mixing one part of 2% EDTA solution (ethylenediaminetetraacetic acid disodium salt; Merck KGaA, Darmstadt, Germany) with nine parts of whole blood, and analyzed using an Element HT5+ hematology analyzer (Heska, Loveland, CO, USA). The following hematology parameters were measured: White blood cell (WBC) count, lymphocyte count, monocyte count, neutrophil count, basophil count, and eosinophil count. The red blood cell indices included the red blood cell (RBC) count, hematocrit, mean corpuscular volume (MCV), and mean corpuscular hemoglobin (MCH). The reticulocyte data included the absolute reticulocyte count. The platelet parameters included the platelet count, mean platelet volume (MPV), and plateletcrit (PCT).

### 2.7. Measurement of Total IgG in Rat Plasma (ELISA)

Total rat IgG was quantified by ELISA on 96-well high-binding plates (Nunc Immuno Plate MaxiSorp, Thermo Fisher Scientific, Waltham, MA, USA). The reagents were from a rat IgG ELISA kit (Invitrogen, Thermo Fisher Scientific, Waltham, MA, USA): capture antibody (1:250 in 1× coating buffer), coating buffer (10×; diluted 1:10), Assay Buffer A (20×; 1:10 for blocking and 1:20 for sample/detection buffer), rat IgG standard (1.6–100 ng/mL), detection antibody (1:250), and TMB chromogen (ready to use). The washes used PBS T (Pierce™ 20X PBS Tween™ 20 Buffer; Thermo Fisher Scientific, Waltham, MA, USA). The concentrated reagents were equilibrated to room temperature and gently warmed; the antibody stock concentrations were verified by lot prior to dilution. The plates were coated with capture antibody (100 µL/well) and incubated for 18 h at room temperature, washed 3 times, blocked (Assay Buffer A 1:10; 200 µL/well), incubated for 2 h at room temperature, washed 3 times, then loaded with standards, blanks, and appropriately diluted samples in duplicate (100 µL/well), incubated for 2 h at room temperature, and washed 3 times. Detection antibody was applied (100 µL/well) and incubated for 1 h at room temperature; then plates were washed 3 times, developed with TMB (100 µL/well; 15 min; RT; dark), and stopped with Stop Solution POD (0.25 M (0.5 N) H_2_SO_4_; prepared 1:4 from 1 mol/L (2 N) AVS TITRINORM sulfuric acid (VWR International GmbH, Darmstadt, Germany)). Absorbance at 450 nm was measured using an Epoch 2 microplate spectrophotometer (Agilent BioTek, Winooski, VT, USA). Concentrations were calculated by four-parameter logistic (4PL) curve fitting and adjusted for sample dilution.

### 2.8. Pathology and Organ Weight

A full necropsy of each animal was performed. All macroscopic findings were noted. The liver, kidneys, adrenals, testes, epididymides, thymus, spleen, brain, and heart of all animals, as well as the seminal vesicle and prostate of male animals, were trimmed of any adherent tissue and their wet weights were taken. The following tissues were fixed in 10% buffered formalin (Morphisto, Offenbach am Main, Germany): All gross lesions, brain, spinal cord, stomach, small and large intestines (including Peyer’s patches), liver, kidneys, adrenals, spleen, heart, thymus, thyroid, trachea and lungs, urinary bladder, lymph nodes (inguinal and mesenterial), sciatic nerve, skeletal muscle, and femur with bone marrow of all animals; testis, epididymides, prostate and seminal vesicles with coagulating glands of male animals; and ovaries uterus, cervix, and vagina in female animals. Tissue sections were then embedded in paraffin and routinely processed for histology, cut into 5 µm sections, and stained with hematoxylin and eosin (HE). Histopathological assessment was performed by an experienced pathologist. Morphological changes were diagnosed based on the INHAND (International Harmonization of Nomenclature and Diagnostic Criteria for Lesions in Rats and Mice) criteria.

### 2.9. Assessment of Operational Workload

To obtain a first descriptive estimate of the husbandry workload associated with the two housing systems, we performed a work assessment during one routine cage-cleaning cycle for the cohort of 10 rats per housing condition. Before data collection, the cleaning workflow was divided into two task blocks. The first comprised husbandry-room tasks in the animal area, including transferring rats between soiled and clean cages, attaching new water bottles, refilling food hoppers, and adding fresh bedding, nesting material, and enrichment. The second block comprised cage-wash area preparation, namely, emptying soiled cages, washing them, refilling clean cages with bedding and nesting material, and staging the prepared cages for return to the animal room. On a routine cleaning day, one experienced animal-care technician recorded the duration of each task block for each housing condition. Measurements were performed once per housing condition (one cleaning cycle per 10-rat cohort) and were therefore interpreted descriptively; no statistics were applied. For reporting, we summarized the total time per cleaning cycle.

### 2.10. Anesthesia and Euthanasia

Animals were anesthetized by intraperitoneal injection of ketamine hydrochloride (Ketamin, CP-Pharma Handelsgesellschaft mbH, Burgdorf, Germany; 100 mg/kg) and xylazine hydrochloride (Xylavet^®^, CP-Pharma Handelsgesellschaft mbH, Burgdorf, Germany; 5 mg/kg). They were euthanized by final blood withdrawal. Blood samples were collected to conduct further analysis.

### 2.11. Statistical Analysis

Statistical analysis was conducted using GraphPad Prism (version 9.1.1, GraphPad Software, LLC, Boston, MA, USA). Continuous outcomes measured once per animal (e.g., Elevated Plus Maze and Open Field Test parameters, clinical chemistry, blood gases and organ weights) were compared between colony- and conventionally housed rats within each sex using unpaired two-tailed Student’s *t*-tests with Welch’s correction for unequal variances. For longitudinal outcomes (body weight, hematology, and IgG over time), only within-timepoint comparisons were conducted, and a simple two-sample Welch t-test was chosen to compare groups, as it allows for heteroskedasticity in the estimation of the pooled variance.

A *p*-value < 0.05 was considered statistically significant.

## 3. Results

### 3.1. Body Weight (Females and Males)

**Figure 2:**
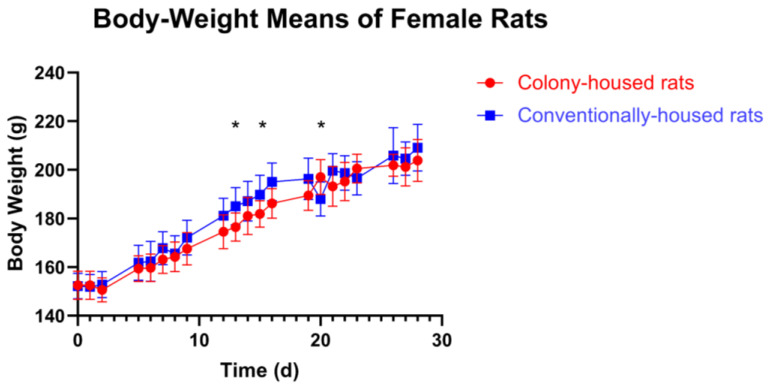
Body-weight means of female Sprague-Dawley rats in two housing conditions during a 28-day study. Body weight (g) measured on working days from day 0 to day 28. Points show group means ± SD; lines connect means across time. Statistical methods are described in Section 2.11. Significance symbols: * *p* < 0.05.

**Figure 3:**
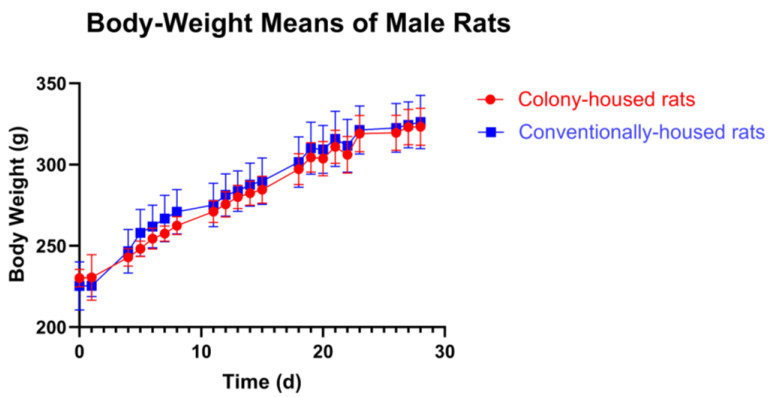
Body-weight means of male Sprague-Dawley rats in two housing conditions during a 28-day study. Body weight (g) measured on working days from day 0 to day 28. Points show group means ± SD; lines connect means across time. Statistical methods are described in Section 2.11.

Across the 28-day study, housing had no consistent effect. In females (Figure 2), most days showed no significant between-group difference (*p* > 0.05); three isolated time points differed: day 13 (conventionally-housed 185.0 g vs. colony-housed 176.4 g; *p* = 0.0137), day 15 (189.8 g vs. 181.9 g; *p* = 0.0209), and day 20 (colony-housed 197.0 g vs. conventionally-housed 188.0 g; *p* = 0.0112). In males, body weight did not differ at baseline or in any subsequent assessment (all *p* > 0.05; Figure 3).

### 3.2. Food and Water Intake

Food and water were recorded at the cage level and summarized by housing condition (colony cages: one cage with 10 same-sex rats; conventional cages: five pair-housed cages; *n* = 10/sex/condition). Females. Baseline body weight (BW, mean ± SD) was 152.5 ± 5.8 g in colony-housed rats and 152.2 ± 5.2 g in conventionally housed rats; terminal BWs were 203.9 ± 8.6 g and 209.1 ± 9.6 g, respectively. Over 28 days, cumulative food intake was 4611.43 g (colony-housed rats) and 4145.64 g (conventionally housed rats), corresponding to 16.47 vs. 14.81 g/rat/day (≈11.24% higher in colony housed rats). Cumulative water intake was 5765.60 mL (colony-housed rats) and 5979.15 mL (conventionally housed rats), i.e., 20.59 vs. 21.35 mL/rat/day (≈3.70% higher in conventionally housed rats).

Males. Baseline BWs were 230.2 ± 5.4 g (colony-housed rats) and 225.3 ± 14.7 g (conventionally housed rats); terminal BWs were 323.3 ± 11.4 g and 326.2 ± 16.4 g, respectively. Over 28 days, cumulative food intakes were 5902.46 g (colony-housed rats) and 6204 g (conventionally housed rats), corresponding to 21.08 vs. 22.16 g/rat/day (≈5.11% higher in conventionally housed rats). Cumulative water intakes were 6966 mL (colony-housed rats) and 7095 mL (conventionally housed rats), i.e., 24.88 vs. 25.34 mL/rat/day (≈1.85% higher in conventionally housed rats).

### 3.3. Behavior Tests

#### 3.3.1. Open Field Test

Across both sexes, conventional housing was associated with greater locomotor activity than colony housing.

**Figure 4:**
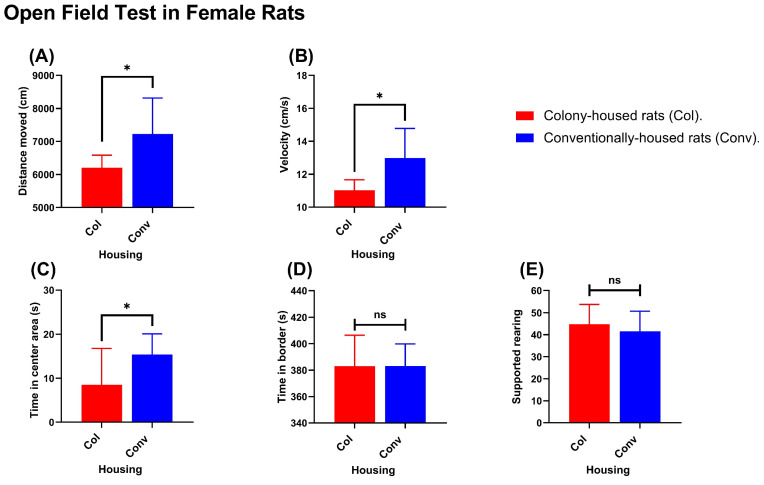
Open Field Test (females): Conventionally housed rats (blue) vs. colony-housed rats (red). Panels show (**A**) total distance moved, (**B**) mean velocity, (**C**) time in the center zone, (**D**) time in the border zone, and (**E**) supported rearing count. Values shown are group means. Significance symbols: * *p* < 0.05; ns, not significant.

**Figure 5:**
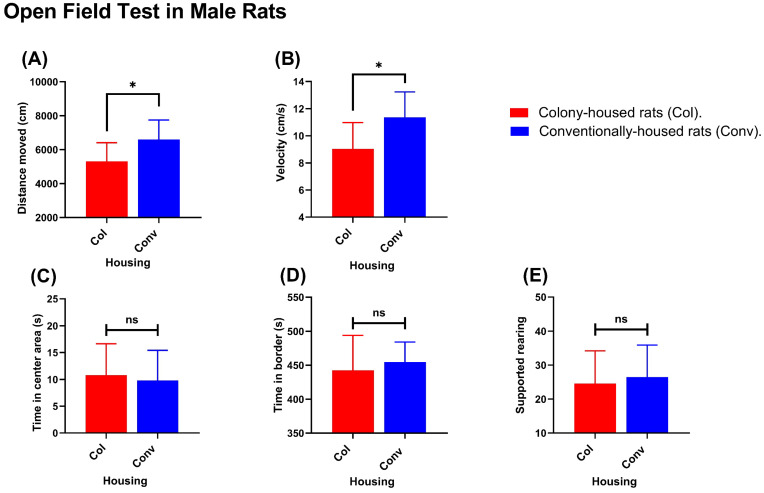
Open-field test (males): Conventionally housed rats (blue) vs. colony-housed rats (red). Panels show (**A**) total distance moved, (**B**) mean velocity, (**C**) time in the center zone, (**D**) time in the border zone, and (**E**) supported rearing count. Values shown are group means. Significance symbols: * *p* < 0.05; ns, not significant.

Females. Conventionally housed females traveled a greater distance than colony-housed females (7227 cm vs. 6202 cm; *p* = 0.0235; Figure 4A). Average velocity was higher in the conventional group (12.99 cm/s vs. 11.03 cm/s; *p* = 0.0113; Figure 4B). Time spent in the center was greater in conventionally vs. colony-housed rats (15.41 vs. 8.518; *p* = 0.0398; Figure 4C). Time spent on the border (383.1 s in conventionally housed rats vs. 380.0 s in colony-housed rats; *p* = 0.9910; Figure 4D) and supported rearing (41.56 events in conventionally housed rats vs. 44.80 events in colony-housed rats; *p* = 0.4463; Figure 4E) did not differ between colony- and conventionally housed animals. Males. Conventionally housed males also showed higher locomotor activity. In the Open Field Test, they traveled a greater distance (6593 cm vs. 5306 cm; *p* = 0.0204; Figure 5A) and had higher velocity (11.36 cm/s vs. 9.03 cm/s; *p* = 0.0137; Figure 5B). Time spent in the center area (9.811 s in conventionally housed rats vs. 10.79 s in colony-housed rats; *p* = 0.7083; Figure 5C), time spent in the border (454.7 s in conventionally housed rats vs. 442.5 s in colony-housed rats; *p* = 0.5266; Figure 5D), and supported rearing frequency (26.5 events in conventionally housed rats vs. 24.6 events in colony-housed rats; *p* = 0.6603; Figure 5E) showed no significant differences between housing conditions.

#### 3.3.2. Elevated Plus Maze Test

**Figure 6:**
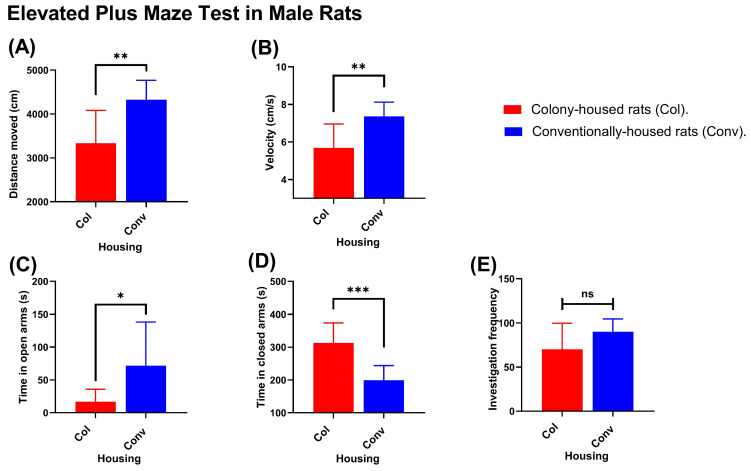
Elevated Plus Maze (EPM) outcomes of male rats housed in conventional (blue) vs. colony (red) conditions. Panels depict (**A**) total distance traveled, (**B**) average velocity, (**C**) time spent in closed arms, (**D**) time spent in open arms, and (**E**) open-arm investigation frequency; *n* = 10 per housing condition. Values shown are group means. Significance symbols: * *p* < 0.05; ** *p* < 0.01; *** *p* < 0.001; ns, not significant.

**Figure 7:**
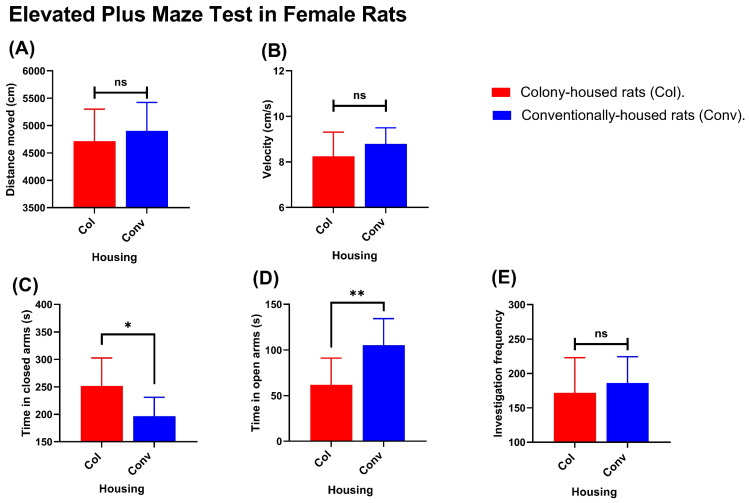
Elevated Plus Maze (EPM) outcomes of female rats housed in conventional (blue) vs. colony (red) conditions. Panels depict (**A**) total distance traveled, (**B**) average velocity, (**C**) time spent in closed arms, (**D**) time spent in open arms, and (**E**) open-arm investigation frequency; *n* = 10 per housing condition. Values shown are group means. Significance symbols: * *p* < 0.05; ** *p* < 0.01; ns, not significant.

Significant differences were observed between housing conditions in measures of locomotor activity and distribution of time in the open and closed arms. In males, conventionally housed rats traveled greater distances than their colony-housed counterparts (4326 vs. 3335 cm; *p* = 0.0027; Figure 6A) and moved faster (7.36 vs. 5.69 cm/s; *p* = 0.0028; Figure 6B). They also spent less time in the closed arms (198.9 vs. 313.0 s; *p* = 0.0002; Figure 6C) and more time in the open arms (71.7 vs. 16.8 s; *p* = 0.0301; Figure 6D). The number of open-arm investigations (entries/explorations) did not differ significantly (90.1 vs. 70.1; *p* = 0.0773; Figure 6E). In females, distance traveled (4906 cm in conventionally housed rats vs. 4714 cm in colony-housed rats; *p* = 0.4492; Figure 7A) and movement velocity (8.788 cm/s in conventionally housed rats vs. 8.248 cm/s in colony-housed rats; *p* = 0.1982; Figure 7B) did not differ between housing conditions, and open-arm investigations were likewise not different (186.3 events in conventionally housed rats vs. 171.8 events in colony-housed rats; *p* = 0.4821; Figure 7E). However, conventionally housed females spent significantly less time in the closed arms than colony-housed females (196.8 vs. 251.8 s; *p* = 0.012; Figure 7) and more time in the open arms (105.2 vs. 61.96 s; *p* = 0.0039; Figure 7D).

### 3.4. Clinical Chemistry and Blood Gases

Comprehensive clinical chemistry and blood gas analysis (BGA) showed that, across both sexes, most parameters did not differ between colony- and conventionally housed rats. **Females**. The only statistically significant differences in females were lower creatinine and sodium in the conventional group (creatinine: 26.8 vs. 30.4 µmol/L; *p* = 0.0306; Na^+^: 145.6 vs. 149.9 mmol/L; *p* = 0.0172).

All other female measures, including liver enzymes (ALT, AST, ALP), serum proteins (albumin, total protein), urea, lipid profiles (total cholesterol, triglycerides), metabolic indices (glucose, lactate), pH and blood gases (pCO_2_, pO_2_), bicarbonate (HCO_3_^−^), base excess (blood and extracellular fluid), oxygen saturation (sO_2_), electrolytes other than sodium (K^+^, Ca^2+^, Cl^−^), phosphate, hematocrit, and calculated hemoglobin were comparable between housing conditions.

Males. No parameter differed significantly between colony and conventional housing; liver enzymes (ALT, AST, ALP), kidney function markers (urea, creatinine), serum proteins, lipid profiles, metabolic indices, pH and blood-gas values, bicarbonate, base excess, sO_2_, electrolytes (Na^+^, K^+^, Ca^2+^, Cl^−^), phosphate, hematocrit, and calculated hemoglobin were comparable between housing conditions (for all non-significant comparisons, *p* > 0.05).

### 3.5. Hematology

**Figure 8:**
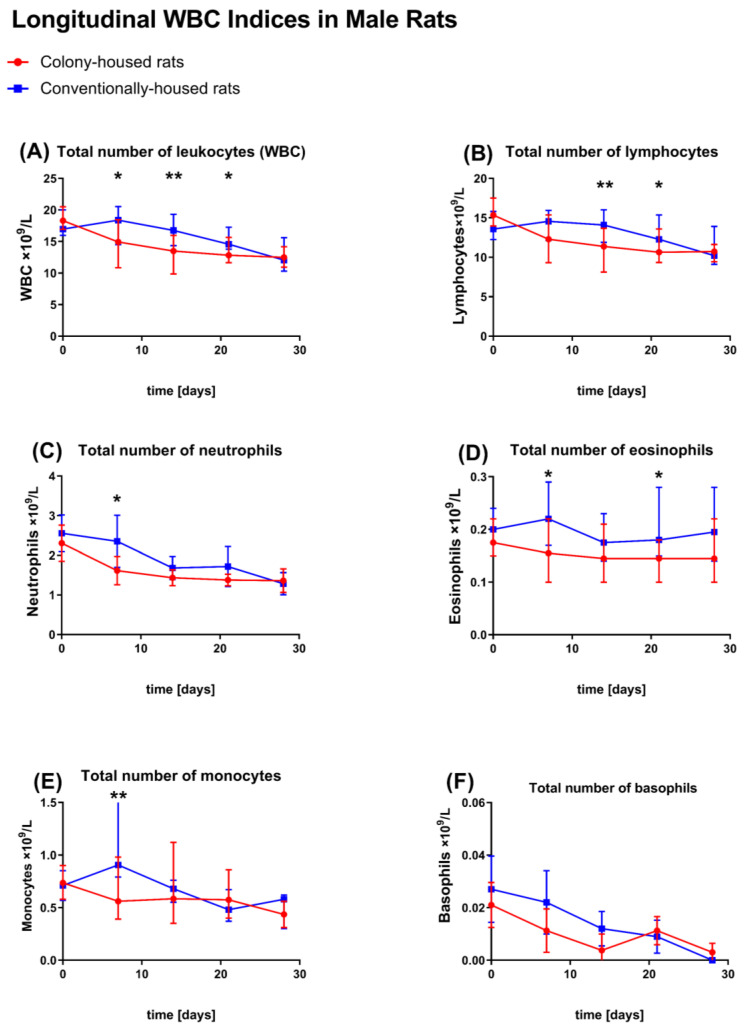
Hematology—longitudinal white blood cell (WBC) indices in males. Conventional (blue squares) vs. colony (red circles), colony *n* = 10 (day 0), *n* = 8 (days 7–21), *n* = 10 (day 28); conventional *n* = 10 at all time points; points show group means; error bars show SD. Blood was collected from the lateral tail vein on days 0, 7, 14, 21, and 28. Units for all panels are ×10^9^/L. (**A**) Total leukocytes (WBC), (**B**) lymphocytes, (**C**) neutrophils, (**D**) eosinophils, (**E**) monocytes, and (**F**) basophils. Asterisks over time points indicate significant between-housing differences within each panel (Significance symbols: *p* < 0.05 = *, *p* < 0.01 = **).

**Figure 9:**
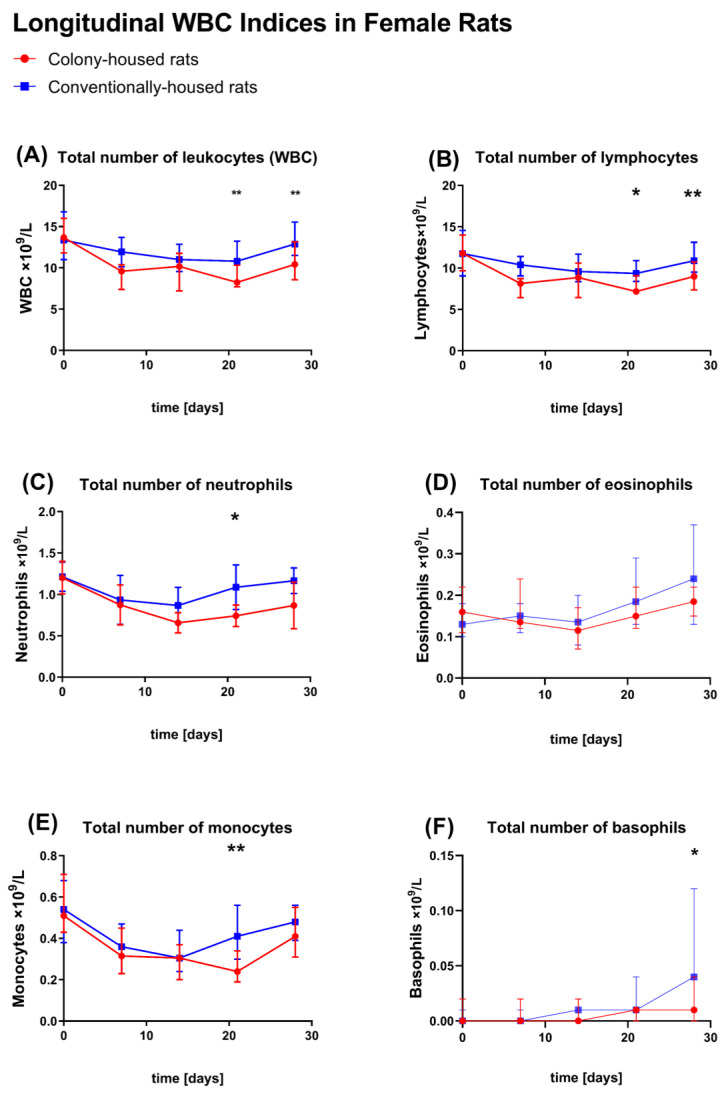
Hematology—longitudinal white blood cell (WBC) indices in females. Conventional (blue squares) vs. colony (red circles), *n* = 10/group; points show group means; error bars show SD. Blood was collected from the lateral tail vein on days 0, 7, 14, 21, and 28. Units for all panels are ×10^9^/L. (**A**) Total leukocytes (WBC), (**B**) lymphocytes, (**C**) neutrophils, (**D**) eosinophils, (**E**) monocytes, and (**F**) basophils. Asterisks over time points indicate significant between housing differences within each panel (Significance symbols: * *p* < 0.05, ** *p* < 0.01.

Baseline (study day 0). Hematologic profiles were largely comparable between housing conditions (*p* > 0.05 for most comparisons) for male (Figure 8A–F) and female (Figure 9A–F) animals. Males. No statistically significant differences were observed between housing conditions for any measured parameter (all *p* > 0.05). **Females**. Conventionally housed animals showed a slightly lower MCV (59.22 vs. 60.12 fL; *p* = 0.0454); no statistically significant differences were observed between housing conditions for any other measured parameter (all *p* > 0.05).

Study day 7. Males. The conventionally housed group exhibited a higher total WBC than the colony-housed group (17.88 vs. 15.01 × 10^3^/μL; *p* = 0.0311), driven by higher neutrophil (2.35 vs. 1.62 × 10^3^/μL; *p* = 0.0422), monocyte (1.02 vs. 0.60 × 10^3^/μL; *p* = 0.0052), and eosinophil counts (0.216 vs. 0.160 × 10^3^/μL; *p* = 0.0304). Lymphocyte and basophil counts were similar (*p* > 0.05). RBC indices were unchanged (RBC 7.00 vs. 7.07 × 10^6^/μL; hemoglobin 14.35 vs. 14.65 g/dL; hematocrit 42.31% vs. 42.73%; MCV/MCH/*p* > 0.05), and platelet parameters were similar (all *p* > 0.05). The reticulocyte count was higher in conventionally housed males (absolute 329.2 vs. 291.7 × 10^3^/μL; *p* = 0.0337). Females. On day 7, conventionally housed animals showed lower MCH (20.11 vs. 20.68 pg; *p* = 0.0355); there were no statistically significant differences in any other hematological parameter between the colony- and conventionally housed groups (*p* > 0.05).

Study day 14. Males. Total WBC remained higher in the conventionally housed group (16.55 vs. 13.05 × 10^3^/μL; *p* = 0.0076), primarily due to an elevated lymphocyte count (14.00 vs. 10.84 × 10^3^/μL; *p* = 0.0048). The neutrophil, monocyte, eosinophil, and basophil counts no longer differed (*p* > 0.05). The RBC indices and reticulocyte absolute counts were comparable between housing conditions (all *p* > 0.05). Females. There were no statistically significant differences in any measured hematological parameter between the colony- and conventionally housed groups (*p* > 0.05).

Study day 21. Males. The conventional group again showed higher total WBC (15.47 vs. 13.28 × 10^3^/μL; *p* = 0.0349), with higher lymphocyte (13.03 vs. 11.14 × 10^3^/μL; *p* = 0.0478) and eosinophil counts (0.204 vs. 0.148 × 10^3^/μL; *p* = 0.0227). RBC parameters, reticulocyte indices, and platelet-related parameters remained equivalent (*p* > 0.05 for all). Females. Conventionally housed animals exhibited a higher total WBC (11.26 vs. 9.03 × 10^3^/μL; *p* = 0.0082), driven by higher neutrophil (1.09 vs. 0.74 × 10^3^/μL; *p* = 0.0220), lymphocyte (9.55 vs. 7.88 × 10^3^/μL; *p* = 0.0313), and monocyte counts (0.42 vs. 0.25 × 10^3^/μL; *p* = 0.0013). In females, MCV was higher in conventionally housed animals than in colony-housed animals (59.43 vs. 57.46 fL; *p* = 0.0010). Among platelet measures, MPV was lower in conventionally housed females (5.42 vs. 5.64 fL; *p* = 0.0152); there were no statistically significant differences in any other hematological parameter between the colony- and conventionally housed groups (*p* > 0.05).

Study day 28. Males. No statistically significant differences were observed in any hematological parameter between housing conditions. Females. Conventionally housed animals again had a higher total WBC (13.19 vs. 10.54 × 10^3^/μL; *p* = 0.0055), primarily reflecting higher absolute lymphocyte counts (11.23 vs. 9.05 × 10^3^/μL; *p* = 0.0070). Basophils were also increased (0.050 vs. 0.010 × 10^3^/μL; *p* = 0.0102); there were no statistically significant differences in any other hematological parameter between the colony- and conventionally housed groups (*p* > 0.05) in female animals.

### 3.6. Endogenous IgG

**Figure 10:**
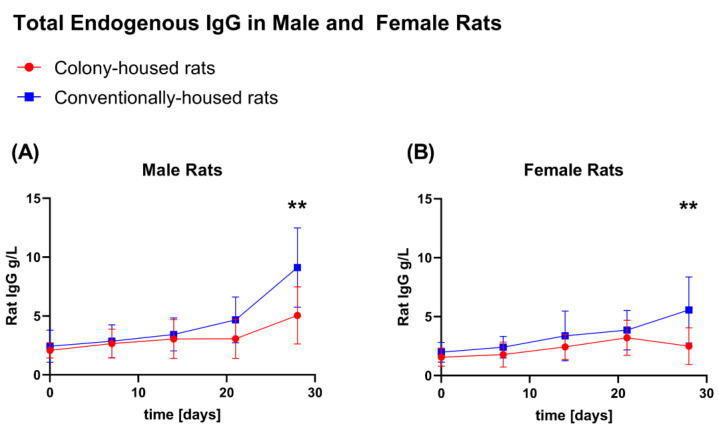
Total endogenous IgG over 28 days in colony- vs. conventionally housed Hsd:Sprague-Dawley rats. IgG (g/L) was quantified from lateral-tail-vein blood at baseline (day 0) and on days 7, 14, 21, and 28. Points show group means; error bars indicate SD. Red circles = colony; blue squares = conventional. (**A**) Males; (**B**) females. Sample sizes: males: colony *n* = 10 (day 0), *n* = 8 (days 7–21), *n* = 10 (day 28); conventional *n* = 10 at all time points. Females: *n* = 10 per group at all time points. Asterisks mark significant between-housing differences at the indicated day (** *p* < 0.01).

Endogenous IgG was quantified on days 0, 7, 14, 21, and 28. Across both sexes, a significant difference was detected only at the terminal time point (day 28; Figure 10A,B), with higher IgG in conventionally housed rats for males (9.121 vs. 5.038 g/L; *p* = 0.0065) and females (5.568 vs. 2.491 g/L; *p* = 0.0088). At earlier time points (days 0, 7, 14, and 21), IgG did not differ by housing condition in either sex (all *p* > 0.05).

### 3.7. Gross Necropsy

No macroscopic lesions were observed at necropsy in any group (except two animals with tail injury due to the misconstruction in the cage). There were no signs of trauma due to fighting or any aggression-related injuries.

### 3.8. Organ Weights

Absolute and relative organ weights (organ-to-body weight ratios in %) are listed separately by sex; males in Table 1 and females in Table 2. No statistically significant differences were observed between housing conditions for any organ listed in these tables in either sex (all *p* > 0.05).

### 3.9. Histopathology

Histopathological assessment revealed no differences between conventionally and colony-housed rats. All diagnosed findings are well-described strain-related background findings in Hsd:Sprague-Dawley rats and not associated with the different housing conditions or a specific treatment. No signs of inflammatory or degenerative processes were observed.

### 3.10. Operational Workload

For the 10-rat cohort, routine husbandry procedures took longer in the colony housing than in conventional pair housing. The total staff time per cage-cleaning cycle was approximately 35 min for a single two-level colony cage housing all 10 rats versus approximately 20 min for five Type IV cages housing pairs of rats. Time spent on husbandry-room tasks (transferring animals, attaching new water bottles, refilling food hoppers, and adding enrichment) was approximately 15 vs. 10 min, and preparation in the cage-wash area (emptying, washing and refilling cages, staging clean cages) required approximately 20 vs. 10 min in colony vs. conventional housing, respectively (Table 3).

## 4. Discussion

In the present study the effect of enriched housing conditions on key parameters in studies performed in early drug development and safety assessment for drugs and chemicals were assessed. To minimize variability in measured experimental parameters, standardization of experimental conditions, such as housing, hygiene management, and the genetic background of the animals is commonly applied. However, the findings of the present study indicate that the most relevant outcome measures remain unaffected by housing conditions, thereby supporting the implementation of refined housing strategies that promote social enrichment without compromising data integrity.

Relevant significant differences between housing conditions were observed in behavioral tests, hematological parameters, and IgG levels. Additionally, significant body weight differences in females at three isolated time points (days 13, 15, and 20) were interpreted as incidental and not related to the housing conditions. At later time points, no significant changes in female body weights were detected, and the weight range at all time points remained within the physiological range for Hsd:Sprague-Dawley rats.

In the behavioral tests (Elevated Plus Maze and Open Field Test), significant differences were found in parameters primarily related to locomotor activity. Conventionally housed animals covered more distance and moved faster than colony-housed animals in both the EPM and the OFT. In the EPM they also spent less time in the closed arms and more time in the open arms, yet the number of open-arm investigations did not differ between groups. In the OFT, exploration of the center area was higher in females of the conventional housing group, whereas males showed no significant between-housing difference in time spent in the center zone of the Open Field Test. Time spent in the border zone of the OFT was comparable between groups for both males and females, and rearing frequency was not affected by housing condition. Although increased open-arm time is often interpreted as reduced anxiety [18], there are also sources that emphasize the role of other parameters, noting that increased locomotor activity could account for more time spent in the open arms of the Elevated Plus Maze [19,20]. Notably, stress itself can elevate locomotor activity in standard behavioral tests [21], which could also explain the increased time spent in the open arms of the Elevated Plus Maze test. The pattern of the locomotor activity effect aligns with prior evidence that environmental enrichment can reduce novelty-induced locomotion in open arenas [22], whereas partial social isolation or simpler housing can produce hyperlocomotion [23]. Colony housing may also introduce social dynamics (dominance, resource competition) that, if unmanaged, elevate stress, particularly in mixed-sex settings [24]. In this study, rats were housed in same-sex groups, males with males and females with females, with ad libitum access to food and water. Throughout acclimation and the active phase, and routine clinical welfare checks (qualitative, anecdotal, non-scored observations by the animal-care staff and attending veterinarian), we observed no aggression, resource guarding, or restricted access to any cage area, arguing against overt social stress as a driver of the behavioral phenotype. Overall, commonly used assays, such as the Open Field Test and the Elevated Plus Maze test, measure parameters like anxiety-like behavior, exploratory activity, and CNS-related effects, including hyperactivity or sedation. These tests’ endpoints can vary significantly depending on factors like illumination, novelty, prior handling, and the animal’s age or strain. This variability makes the tests highly sensitive to the animal’s internal state, which is valuable for revealing individual differences in condition. However, interpretation should be tempered by an awareness of their limitations and by the fact that these assays may be artificial and do not necessarily reflect the behavior of animals in nature [25]. In the context of drug development or safety assessment, behavioral tests are often used to assess potential central nervous system effects of drugs or in the context of developmental neurotoxicity in chemical safety assessments, where chronic exposure to a chemical may influence brain development [26]. In both experimental conditions, the effects observed in the EPM and OFT are expected to be more pronounced than the behavioral differences attributable to variations in housing conditions. In the present study, housing-related differences in the OFT and EPM were limited, primarily involving novelty-induced locomotion. Thus, colony housing can likely be implemented without compromising these standard behavioral endpoints but it may attenuate novelty-driven hyperactivity. Importantly, because healthy animals were assessed under two primarily non-harmful housing conditions, the observed differences need not signal abnormality; rather, they likely reflect a wider, species-typical range of coping styles and exploratory tendencies. In other words, the small shifts we detected are consistent with normal behavioral diversity variation that is expected in rats and does not, on its own, indicate adverse effects or pathology.

Turning to hematology, profiles were broadly comparable across housing conditions; Baseline hematology was comparable across housing. In males, conventional housing induced a transient leukocytosis: Higher WBC at day 7 (neutrophils, monocytes, eosinophils), persisting at day 14 (lymphocytes) and day 21 (lymphocytes and eosinophils), then resolving by day 28. Females showed no differences up to day 14; at days 21 and 28, conventionally housed animals had higher total WBC, driven by neutrophils, lymphocytes, and monocytes at day 21, and by lymphocytes and basophils at day 28. RBC and platelet indices were otherwise similar and within expected ranges. IgG did not differ at interim time points but was higher at day 28 under conventional housing in both sexes.

A plausible explanation for the hematology differences is the higher opportunity for spontaneous daily activity in the colony cages (climbing, exploring, social interaction). Frodermann et. al. (2019) [27] described how higher physical activity can modulate hematopoiesis, shifting hematopoietic stem and progenitor cell proliferation, reducing the output of pro-inflammatory leukocytes, and dampening the systemic inflammatory tone, and could therefore account for the lower total white blood cell counts and the altered leukocyte distribution observed in colony-housed rats in our dataset. Prior work also suggests that housing design details can influence leukocyte profiles: Multilevel systems with restricted access have been associated with higher neutrophils and a higher neutrophil-to-lymphocyte ratio compared with setups that allow unrestricted access [28]. In our study, colony cages provided unrestricted movement between levels, which may have mitigated such effects. Other factors could contribute as well. Acute stress has been reported to transiently elevate circulating immunoglobulins, including total IgG [29], which might help explain some of the IgG differences we observed at the terminal time point. Infection or hygiene status is unlikely to be a driver here: All animals (colony- and conventionally housed) were housed in the same room under identical hygiene management and specific-pathogen-free (SPF) conditions, and neither histopathology nor clinical chemistry indicated inflammation or infection.

Conclusions regarding the statistical significance of the parameters assessed in this study should be interpreted within the context of the sample size and the associated statistical power. We selected a group size of 10 rats per sex and housing condition to align with typical repeated-dose toxicity studies and to limit animal use. Consequently, non-significant results should be interpreted cautiously, particularly for small effect sizes, and future studies could use a priori power analyses, as recommended by Gaskill and Garner (2020) [30], to optimize sample size while adhering to the 3Rs.

Follow-up studies could clarify the mechanisms responsible for the changes seen in behavior tests as well as hematology and IgG levels by measuring fecal corticosterone metabolites and serum corticosterone [31,32]; quantifying serotonin and dopamine in defined brain regions [33,34] to index stress physiology; and performing immune phenotyping (e.g., flow cytometry of leukocyte subsets and IgG subclass profiling) at multiple time points. These targeted readouts would help link housing, activity, stress biology, and immune function more directly.

Complementing the biological outcomes, we recorded staff time for routine husbandry under each housing condition to estimate operational workload. The time was recorded once per condition. The total staff time for routine husbandry of a 10-rat cohort was longer in the colony cage than in five Type IV cages, approximately 35 vs. 20 min per cleaning cycle, primarily reflecting the additional preparation and cleaning required for the larger enclosure. We view these figures as a baseline rather than a fixed additional workload: with incremental workflow standardization and minor engineering refinements to the colony cage (e.g., modular removable inserts, pre-staged bedding and enrichment, and quick-release water systems), cycle time is expected to decrease, narrowing the observed difference between housing conditions.

## 5. Conclusions

Taken together, enriched colony housing produced only modest differences relative to conventional pair housing. Hematologic shifts were small, largely time-limited (higher leukocytes in conventional housing during weeks 1 to 3), and by study-end either resolved (males) or remained minor in magnitude (females), with values within physiologically reference ranges. Behavioral readouts (EPM/OFT) differed mainly at the level of locomotor activity.

These findings support the implementation of colony housing as a refinement that preserves the stability of key endpoints used in early drug discovery and safety assessment. Once the implementation of colony cages is complete, historical control data should be collected for all relevant key parameters. This data should serve as the basis for assessing treatment-related findings in colony-housed animals. Although the integration of new approach methodologies (NAMs) is expected to become more widespread in future research, in vivo studies will continue to play a critical role in drug development and safety assessment. Therefore, refinement of husbandry systems for laboratory rats remains an essential component in advancing animal welfare standards. We propose colony housing in rats as a valuable contribution in this regard.

## Figures and Tables

**Table 1:** Absolute and relative organ weights in male Hsd:Sprague-Dawley rats after 28 days of colony or conventional housing. Values are group means. Absolute weights are reported in grams; relative weights in percentage are calculated as (organ weight/terminal body weight) × 100 (% BW). Sample sizes (Col; Conv) are shown in the rightmost column; *n* = 10 per group for all organs except brain (*n* = 5 per group).

Organ	Absolute Weight		Relative Weight	
	Colony-Housed Mean	Conventionally Housed Mean	Colony-Housed Mean	Conventionally Housed Mean
Liver	11.6	12.23	3.543	3.697
Right kidney	1.141	1.148	0.3487	0.3476
Left kidney	1.131	1.136	0.3456	0.3439
Right adrenal gland	0.0324	0.02526	0.009833	0.007656
Left adrenal gland	0.0253	0.02621	0.007721	0.007939
Right testicle	1.815	1.809	0.5545	0.5472
Left testicle	1.788	1.855	0.5461	0.5615
Right epididymis	0.5506	0.5701	0.168	0.1728
Left epididymis	0.5322	0.5593	0.1625	0.1693
Prostate + seminal vesicles (with coagulating glands), whole	2.277	2.046	0.6946	0.6198
Thymus	0.4522	0.4844	0.1383	0.1467
Spleen	1.65	0.8921	0.4996	0.2702
Brain	1.77	1.809	0.5498	0.5651
Heart	1.196	1.152	0.3654	0.3479

**Table 2:** Absolute and relative organ weights in female Hsd:Sprague-Dawley rats after 28 days of colony or conventional housing. Values are group means. Absolute weights are reported in grams; relative weights in percentage are calculated as (organ weight/terminal body weight) × 100 (% BW). Sample sizes (colony-housed animals (Col); conventionally housed animals (Conv)) are shown in the rightmost column; *n* = 10 per group for all organs except brain (*n* = 5 per group).

Organ	Absolute Weight		Relative Weight	
	Colony-Housed Mean	Conventionally Housed Mean	Colony-Housed Mean	Conventionally Housed Mean
Liver	7.294	7.475	3.576	3.574
Right kidney	0.6736	0.6832	0.3308	0.3277
Left kidney	0.7287	0.6858	0.3599	0.3289
Right adrenal gland	0.0226	0.0208	0.01119	0.01005
Left adrenal gland	0.0241	0.0226	0.01192	0.01093
Paired ovaries	0.1285	0.1347	0.06328	0.06437
Thymus	0.3167	0.3257	0.155	0.1557
Spleen	0.6303	0.6933	0.3096	0.3312
Brain	1.647	1.631	0.8208	0.8024
Heart	0.7634	0.8044	0.3748	0.3847

**Table 3:** Estimated staff time per routine cage-cleaning cycle for a 10-rat cohort housed in conventional Type IV cages versus a two-level colony cage.

Work Step	Conventional Type IV Cages (10 Rats, 5 Cages Total)	Colony Cages (10 Rats, 2 Levels)
Cleaning in the animal room (transfer)	approx. 10 min	approx. 15 min
Preparation in the cage-wash area	approx. 10 min	approx. 20 min
Total time	approx. 20 min	approx. 35 min

## Data Availability

The original contributions presented in this study are included in the article. Further inquiries can be directed to the corresponding author.
